# Morphological Spectrum of the Lateral Pterygoid Muscle: Radioanatomical Analysis, Systematic Review, and Meta-Analytic Synthesis

**DOI:** 10.3390/medicina61101780

**Published:** 2025-10-01

**Authors:** George Triantafyllou, Panagiotis Papadopoulos-Manolarakis, Nikolaos-Achilleas Arkoudis, Georgios Velonakis, Alexandros Samolis, Katerina Vassiou, Aliki Fiska, Maria Piagkou

**Affiliations:** 1 Department of Anatomy, School of Medicine, Faculty of Health Sciences, National and Kapodistrian University of Athens, 11527 Athens, Greece; georgerose406@gmail.com (G.T.); p.papado89@gmail.com (P.P.-M.); alexsamolis@me.com (A.S.); 2 ”VARIANTIS” Research Laboratory, Department of Clinical Anatomy, Masovian Academy in Płock, 09402 Płock, Poland; 3 Department of Neurosurgery, General Hospital of Nikaia-Piraeus, 18454 Athens, Greece; 4 Research Unit of Radiology and Medical Imaging, School of Medicine, National and Kapodistrian University of Athens, 11527 Athens, Greece; nick.arkoudis@gmail.com (N.-A.A.); gvelonakis@med.uoa.gr (G.V.); 5 Second Department of Radiology, General University Hospital “Attikon”, National and Kapodistrian University of Athens, 12462 Athens, Greece; 6 Department of Anatomy, Faculty of Medicine, University of Thessaly, Biopolis, 41110 Larissa, Greece; avassiou@gmail.com; 7 Laboratory of Anatomy, School of Medicine, Democritus University of Thrace, 68150 Alexandroupolis, Greece; afiska@med.duth.gr

**Keywords:** lateral pterygoid muscle, variation, evidence-based anatomy, radiology

## Abstract

*Background and Objectives*: The lateral pterygoid muscle (LPM) is typically described as a two-headed muscle within the infratemporal fossa. However, cadaveric and imaging studies have revealed substantial variability in the number of heads, insertion patterns, and relations to neurovascular structures. *Materials and Methods*: An observational study of 250 brain computed tomography angiographies (CTAs) was performed to assess LPM morphology. Additionally, a systematic review and meta-analysis were conducted in accordance with PRISMA 2020 and Evidence-based Anatomy guidelines. Pooled prevalence estimates were calculated with random-effects models. *Results*: The current study included 250 CTAs for the original study and 1702 muscles for the meta-analytic evidence. During the original study, the two-headed configuration was most common (74.4%), followed by three-headed (14%), one-headed (10.8%), and four-headed (0.8%) morphologies. Symmetry was observed in 75.2% of patients. Meta-analysis confirmed the predominance of the two-headed type (73.98%, 95% CI: 68.22–79.38), with three-headed (16.82%), one-headed (4.37%), and four-headed (<0.01%) variants occurring less frequently. Subgroup analyses showed no significant differences by study type or sample size, though European populations exhibited a higher prevalence of one-headed forms. *Conclusions*: The LPM demonstrates considerable morphological variability, extending beyond the traditional two-headed model. Recognition of these variants is essential for understanding temporomandibular joint function, interpreting imaging, and planning surgical or interventional procedures within the infratemporal fossa. Advanced imaging provides a reliable tool for individualized anatomical assessment, supporting safer clinical practice.

## 1. Introduction

The infratemporal fossa (ITF) exhibits a highly complex anatomy with typical and variant features that have critical clinical and surgical applications. Numerous variants have been described in its arterial [1], neural [2], osseous [3], and muscular [4] structures.

The masticatory group is composed of four major muscles: the masseter, temporalis, and the pterygoid muscles (lateral pterygoid-LPM and medial pterygoid-MPM). All are innervated by branches of the mandibular nerve (V3)—the third division of the trigeminal nerve (TN) [2,5]. Their action produces movements of the mandible at the temporomandibular joint (TMJ). The ITF contains the pterygoid muscles and the tendon of the temporalis, while the masseter lies superficially along the ramus of the mandible [5].

Classically, the LPM is described as a two-headed muscle, with a superior head (SH) originating from the greater sphenoidal wing—specifically its infratemporal crest—and inserting into the articular disk and capsule of the TMJ, and an inferior head (IH) arising from the lateral plate of the pterygoid process and attaching to the pterygoid fovea [5]. The LPM receives its arterial supply from the maxillary artery (MA), a branch of the external carotid artery, though the LPM–MA relationship varies considerably, with the artery coursing superficial, deep, or occasionally through the muscle [1,6].

Morphological variability of the LPM has been repeatedly reported, involving the number of heads, innervation patterns, and insertion footprints [4,7,8]. However, inconsistencies remain between cadaveric and imaging-based investigations. MRI studies have shown that SH morphology is strongly associated with temporomandibular disk displacement [9]. Beyond structural anatomy, advanced biomechanical and functional studies, such as finite element analysis and speech-related electromyography, underscore that the LPM plays critical roles not only in mastication but also in mandibular kinematics and orofacial function [10,11].

To date, no meta-analytic synthesis has systematically quantified the prevalence of LPM morphologies across populations, while only a few large-scale radiological analyses dedicated to this muscle have used advanced vascular imaging. Computed tomography angiography (CTA), although routinely performed in clinical practice, has been previously used only once with a large sample to assess the morphology of the LPM and its relationship to the MA [6]. Therefore, the current evidence-based study aimed to present meta-analytic evidence of the LPM morphological variability, in addition to our original anatomical data derived from CTAs. By establishing these morphological variations, we aimed to present their clinical significance.

## 2. Materials and Methods

### 2.1. Original Study

Two hundred and fifty (250) brain CTAs were randomly selected and retrospectively analyzed for the LPM variable number of heads and the presence of accessory muscles. The gender distribution included 138 males and 112 females, with a mean age of 59.5 ± 14.5 years.

The scans were performed with a helical high-speed, low-dose scanner (SOMATOM go.Top, 128-slice configuration, Siemens Healthineers, Erlangen, Germany) with the patient’s head in the supine neutral position, following the injection of 60 mL of a 30% iodine solution at a flow rate of 4–4.5 mL/s. The scans were obtained from the General Hospital of Nikaia-Piraeus, having received ethical approval from the appropriate authorities (protocol number: 56485, date of approval: 13.11.2024).

Two authors independently reviewed the files (GTr, PPM), and the other authors resolved any discrepancies. The LPM was identified in sagittal slices with the multiplanar reconstruction mode (simultaneous observation in axial, coronal, and sagittal slices), and the number of heads was determined. The study was conducted and documented utilizing the Horos software (Horos Project, New York, NY, USA).

Statistical analysis was performed with IBM Statistics for macOS, Version 29 (IBM Corp., Armonk, NY, USA). The Chi-square test was used for unpaired nominal data, while McNemar’s test was applied for paired nominal observations. A *p*-value of less than 0.05 was considered statistically significant.

### 2.2. Systematic Review and Meta-Analysis

The systematic review with meta-analysis was performed according to the Evidence-based Anatomy Workgroup guidelines for anatomical meta-analysis [12] and the PRISMA 2020 guidelines for systematic reviews [13], up to June 2025. The protocol of this study was registered in the PROSPERO database under the following number CRD420251149650.

A literature search was performed on the online databases PubMed, Google Scholar, Scopus, and Web of Science. The following terms were used in several combinations: “lateral pterygoid muscle”, “variation”, “cadaveric study”, and “imaging study”. A secondary search was performed with the references of the included articles, the gray literature, and a hands-on search of the major anatomical journals. The search strategy was not restricted by year of publication or language of the studies. Inclusion criteria were studies reporting the prevalence of LPM variants (heads and accessory muscles). Case reports, conference abstracts, animal studies, and studies that reported irrelevant or insufficient data were excluded.

Literature search and extraction into Microsoft Excel sheets were performed by two independent reviewers. The other authors resolved potential disagreements. Risk of bias assessment was evaluated with the Anatomical Quality Assurance (AQUA) tool, proposed by the Evidence-based Anatomy Workgroup for anatomical systematic reviews [14].

Statistical analyses were conducted in RStudio (v4.3.2; RStudio Team, Boston, MA, USA) using the “meta” and “metafor” packages. Pooled prevalence estimates were determined with an inverse variance random-effects model, utilizing the Freeman–Tukey double arcsine transformation. Between-study variance (τ^2^) was estimated via the DerSimonian–Laird method, with its confidence interval calculated using the Jackson method. Heterogeneity was assessed using Cochran’s Q test (*p* < 0.10 considered significant) and quantified with the Higgins I^2^ statistic (0–40%: low; 30–60%: moderate; 50–90%: substantial; 75–100%: considerable). Statistical significance was set at *p* < 0.05. To detect potential small-study effects, a DOI plot with the LFK index was generated [15].

## 3. Results

### 3.1. Original Study

The LPM was identified in all 250 patients (100%).

**Figure 1:**
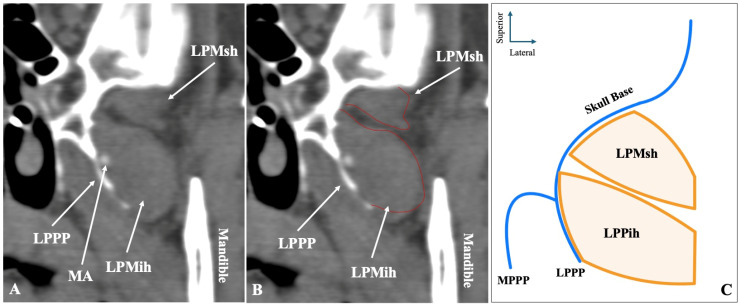
The two-headed configuration of the lateral pterygoid muscle (LPM) observed in sagittal computed tomography angiography (CTA) slices (**A**,**B**) and in schematic representation (**C**). LPMsh = lateral pterygoid muscle, superior head; LPMih = lateral pterygoid muscle, inferior head; LPPP = lateral pterygoid process plate.

The most common morphology of the muscle was the two-headed configuration (superior-inferior) recorded in sagittal slices, identified in 372 sides (74.4%) (Figure 1). Symmetrical two-headed morphology was noted in 152 patients (60.8%). Out of the 372 sides with two-headed LPM, 230 sides (61.8%) had the MA lateral to the muscle, 122 sides (32.8%) medial to the muscle, and 20 sides (5.4%) through it.

**Figure 2:**
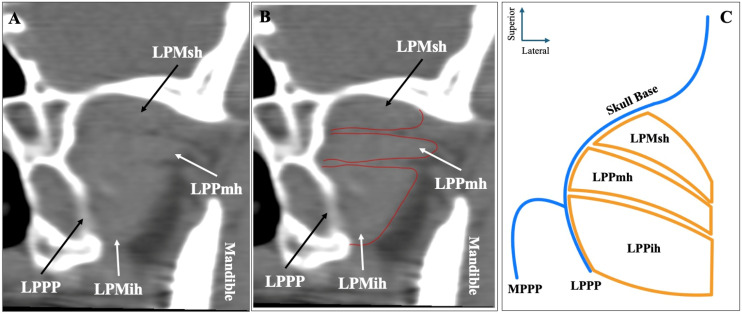
The three-headed configuration of the lateral pterygoid muscle (LPM) is shown in sagittal CTA slices (**A**,**B**) and in schematic representation (**C**). LPMsh = superior head; LPMmh = middle head; LPMih = inferior head; LPPP = lateral pterygoid process plate.

The second most common morphology was the three-headed configuration (superior-middle-inferior) recorded in sagittal slices, observed in 70 sides (14%) (Figure 2). Out of the 70 sides with three-headed LPM, 44 (62.9%) had the MA lateral to the muscle, 16 (22.9%) had the MA medial, and 10 (14.2%) had the artery through the muscle.

**Figure 3:**
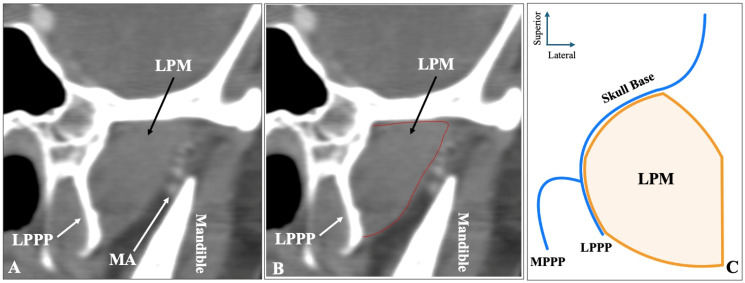
The one-headed configuration of the lateral pterygoid muscle (LPM) recorded in sagittal CTA slices (**A**,**B**) and in schematic representation (**C**). LPPP = lateral pterygoid process plate.

The third most common morphology was the one-headed configuration (no difference between superior-inferior) recorded in sagittal slices, observed in 54 sides (10.8%) (Figure 3). Out of 54 sides with one-headed LPM, 42 sides (77.8%) had the MA lateral, 12 sides (22.2%) had the artery medial to the muscle, and zero patients had the artery through the muscle on this type.

**Figure 4:**
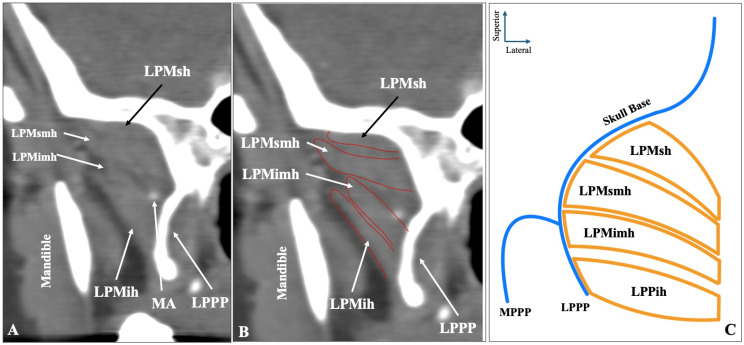
The rare four-headed configuration of the lateral pterygoid muscle (LPM) as visualized in sagittal CTA slices (**A**,**B**) and in schematic representation (**C**). LPMsh = superior head; LPMsmh = superior middle head; LPMimh = inferior middle head; LPMih = inferior head; LPPP = lateral pterygoid process plate.

The rarest configuration was the four-headed muscle (superior-superior, middle-inferior, middle-inferior) observed in sagittal slices, identified four sides (0.8%) (Figure 4). Out of the four sides with four-headed morphology, two cases (50%) had the MA medial to the muscle and two cases (50%) through the muscle.

Descriptive statistics regarding sides and sex distributions are summarized in Table 1. Lastly, it is essential to mention that 188 patients had symmetrical LPM morphology (75.2%) and 62 patients had asymmetrical (24.8%).

### 3.2. Systematic Review

**Figure 5:**
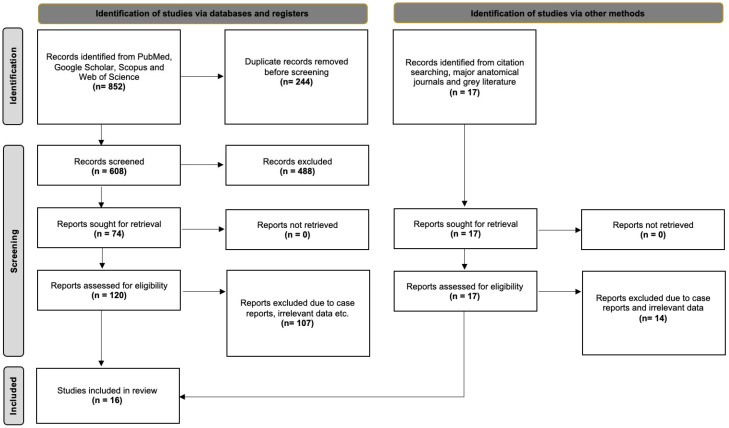
PRISMA 2020 flow diagram illustrating the literature search and study selection process for the systematic review and meta-analysis of lateral pterygoid muscle (LPM) morphology. A total of 852 records were identified through electronic databases, with 244 duplicates removed, leaving 608 records screened. After exclusion of 488 irrelevant records, 120 full-text reports were assessed for eligibility, of which 107 were excluded. Seventeen additional studies were identified through citation searching, gray literature, and hand-searching anatomical journals, of which three were included. In total, 16 studies met the inclusion criteria and were analyzed.

The database search identified 852 articles exported to Mendeley version 2.10.9 (Elsevier, London, UK). Thirteen (13) studies were eligible, and three (3) more studies were identified from our secondary investigation (references, gray literature, and a hands-on search of anatomical journals). Thus, 16 studies were included in our systematic review with meta-analysis. Figure 5 summarizes the flow diagram of our search analysis based on the PRISMA 2020 guidelines.

Sixteen (16) studies with a total sample of 1702 muscles were included in the systematic review and meta-analysis. Thirteen (13) studies were cadaveric, and three (3) were based on imaging techniques. Among these, nine (9) were conducted in Asian populations, four (4) in European populations, and three (3) in American populations. Regarding study size, ten (10) investigations analyzed fewer than 50 muscles, whereas six (6) studies included more than 50 muscles, indicating variable statistical power across studies. The methodological characteristics and risk of bias assessment of the eligible studies are summarized in Table 2.

### 3.3. Meta-Analysis

**Figure 6:**
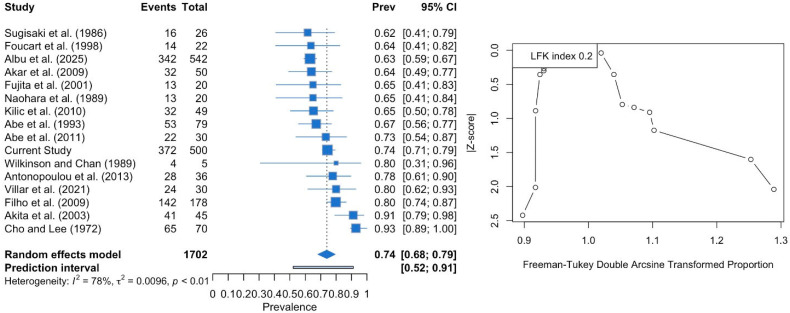
Forest plot and DOI plot for the pooled prevalence of the two-headed lateral pterygoid muscle (LPM). The forest plot (**left**) displays study-specific prevalence estimates with 95% confidence intervals and weights. The pooled prevalence under a random-effects model was 74.0% (95% CI: 68.0–79.0). Significant heterogeneity was detected (I^2^ = 78%, *p* < 0.01). The DOI plot (**right**) shows an LFK index of +0.2, indicating symmetry and no evidence of small-study effects [6,8,16,17,18,19,20,21,22,23,24,25,26,27,28].

The two-headed LPM was estimated with a pooled prevalence of 73.98% (95% CI: 68.22–79.38). The DOI plot depicted an LFK index of +0.2 (no asymmetry), indicating no small-study effect (Figure 6). Similarly, the subgroup analysis of studies with fewer than 50 subjects and those with more than 50 subjects did not show a statistically significant result (*p* = 0.764). The cadaveric studies had a higher prevalence (74.25%) compared to the imaging-based studies (72.41%), although this difference was not statistically significant (*p* = 0.878). Studies performed in the American population yielded higher prevalence estimates (81.64%) compared to Asian (73.51%) and European (69.96%) populations, but again, the difference did not reach statistical significance (*p* = 0.123).

**Figure 7:**
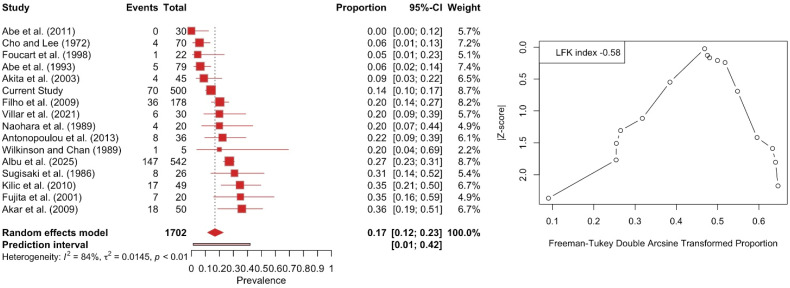
Forest plot and DOI plot for the pooled prevalence of the three-headed lateral pterygoid muscle (LPM). The forest plot (**left**) displays study-specific prevalence estimates with 95% confidence intervals and weights. The pooled prevalence under a random-effects model was 16.8% (95% CI: 11.6–22.8). Considerable heterogeneity was detected (I^2^ = 84%, *p* < 0.01). The DOI plot (**right**) shows an LFK index of −0.58, indicating symmetry and no evidence of small-study effects [6,8,16,17,18,19,20,21,22,23,24,25,26,27,28].

The three-headed LPM was calculated with a pooled prevalence of 16.82% (95% CI: 11.56–22.75). The DOI plot depicted an LFK index of −0.56 (no asymmetry), indicating no small-study effect (Figure 7). Similarly, the subgroup analysis between studies with a sample of fewer than 50 subjects and with more than 50 subjects did not depict a statistically significant result (*p* = 0.8173). Subgroup analysis based on nationality and type of study yielded no statistically significant results (*p* = 0.8197 and *p* = 0.6297, respectively).

**Figure 8:**
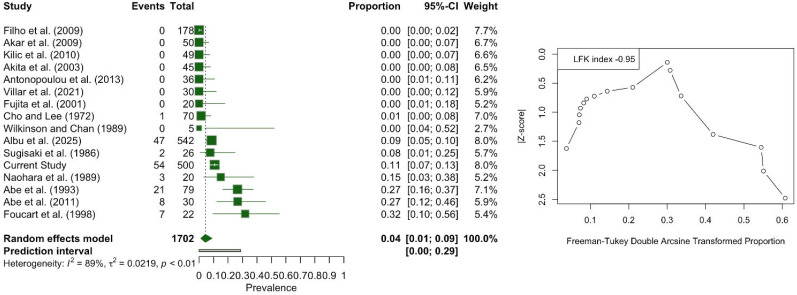
Forest plot and DOI plot for the pooled prevalence of the one-headed lateral pterygoid muscle (LPM). The forest plot (**left**) displays study-specific prevalence estimates with 95% confidence intervals and weights. The pooled prevalence under a random-effects model was 4.0% (95% CI: 1.0–9.0). Substantial heterogeneity was detected (I^2^ = 89%, *p* < 0.01). The DOI plot (**right**) shows an LFK index of −0.95, indicating symmetry and no evidence of small-study effects [6,8,16,17,18,19,20,21,22,23,24,25,26,27,28].

The one-headed LPM was calculated with a pooled prevalence of 4.37% (95% CI: 1.16–8.94). The DOI plot showed an LFK index of −0.95 (no asymmetry), indicating no small-study effect (Figure 8). Similarly, the subgroup analysis of the studies with fewer than 50 subjects and those with more than 50 subjects did not show a significant difference (*p* = 0.9412). Studies conducted in the European population had a pooled prevalence of 8.83%, compared to 5.06% in the Asian population and 5.06% in the American population, with a statistically significant difference (*p* < 0.0001). Subgroup analysis based on study type did not produce statistically significant results (*p* = 0.8140).

**Figure 9:**
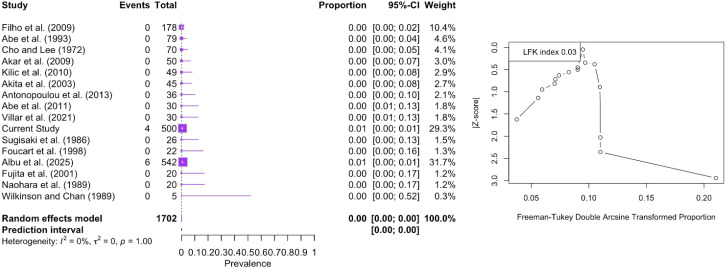
Forest plot and DOI plot for the pooled prevalence of the four-headed lateral pterygoid muscle (LPM). The forest plot (**left**) displays study-specific prevalence estimates with 95% confidence intervals and weights. Only two studies reported cases of this morphology, yielding a pooled prevalence of <0.01% (95% CI: 0.00–0.12) under a random-effects model. No heterogeneity was detected (I^2^ = 0%, *p* = 1.00). The DOI plot (**right**) shows an LFK index of +0.03, indicating symmetry and no evidence of small-study effects [6,8,16,17,18,19,20,21,22,23,24,25,26,27,28].

The four-headed LPM was identified with a pooled prevalence of <0.01% (95% CI: 0.00–0.12). This type was reported only in six cases from the study of Albu et al. [6] and four cases in the current study, out of 1702 muscles. The DOI plot depicted an LFK index of +0.03 (no asymmetry), indicating no small-study effect (Figure 9).

**Figure 10:**
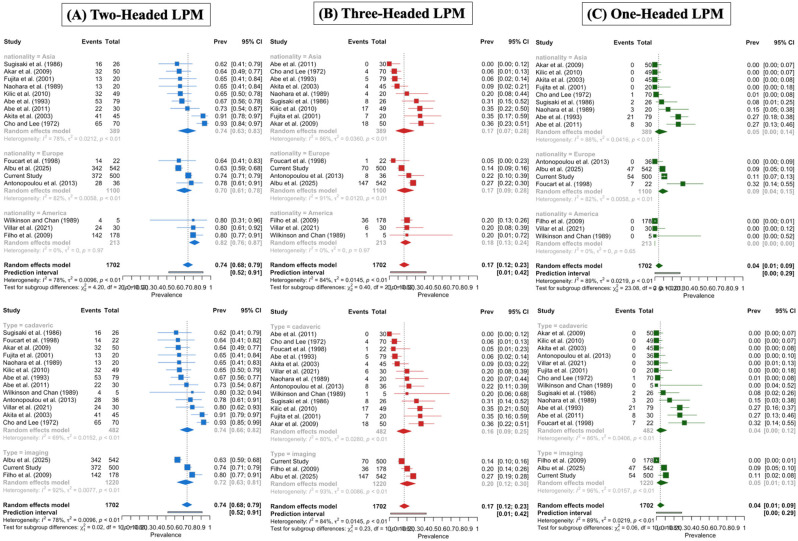
Subgroup analysis based on nationality and type of study for two-, three-, and one-headed lateral pterygoid muscle [6,8,16,17,18,19,20,21,22,23,24,25,26,27,28].

A detailed analysis of the subgroup analysis based on geographical distribution, type of study, and sample size is summarized in Table 3, Figure 10.

## 4. Discussion

The LPM is traditionally described as a two-headed muscle with SH and IH. However, evidence from cadaveric, imaging, developmental, and histological studies suggests much greater complexity. Our radioanatomic and meta-analytic study highlights that the two-headed LPM is present in 73.98% of the population, while the remaining 26.02% display alternative morphologies.

### 4.1. Anatomical Considerations of the Lateral Pterygoid Muscle (LPM) Variability

Embryological research demonstrates that the distinction between the SH and IH of the LPM develops progressively, with the SH appearing later in gestation as an “anterior slip” from the main muscle mass [29]. This developmental trajectory may underline the frequent anatomical variability observed in adults, such as partial separations, fused slips, or accessory heads [29].

Dissection and imaging studies verify that one-, two-, and three-headed morphologies can occur. A systematic review by Stöckle et al. [4] reported that two-headed types are most common (70–90%), with smaller numbers of one-headed (5–15%) and three-headed (5–30%) forms. In our study, the meta-analysis confirmed these pooled values and also showed a significantly higher prevalence of one-headed morphology in European populations. This might reflect subtle population-specific morphogenetic or functional adaptations [4].

Methodological approaches strongly influence the interpretation of LPM divisions. Some authors argue that muscle should be considered a continuous sheet of fibers with regional alterations rather than sharply divided heads [6,7]. This perspective aligns with embryological and innervation evidence, suggesting that the “head” concept may be more functional than strictly anatomical.

Regarding the radiological modality, the current study used CTA, similar to Albu et al. [6], and successfully demonstrated the LPM morphological variability. In a previous MRI study, Filho et al. [25] investigated only the third head of LPM and identified it in 20.22% of their sample, close to the current results. Therefore, all imaging techniques (CTA and MRI) can be valuable to detect the morphology of the LPM.

Beyond the classical descriptions, rarer muscular variants have been documented [30]. The musculus pterygoideus proprius, first described by Henle in 1858, remains the best-known accessory slip. Its prevalence ranges from 0.67 to 4.5% in cadaveric series to ~12.8% in imaging-based studies [31]. Recently, Rusu et al. [32] identified additional accessory bundles such as the maxillomandibularis muscle, expanding the morphologic spectrum of the ITF.

Insertion patterns also vary. While the inferior head consistently inserts into the pterygoid fovea, the SH displays heterogeneous attachments: to the articular disk–capsule complex, the condylar head, or both [8,9]. Some individuals exhibit a subdivided SH, with one slip to the disk and another to the condylar process. MRI studies confirm that SH morphology correlates strongly with TMD displacement, emphasizing its clinical significance [9].

Histological studies further refine our understanding. The SH predominantly contains fatigue-resistant type I fibers, while the IH shows a mixed composition [33]. Later studies reported age-related remodeling, fiber atrophy, and increased type II fiber proportions, particularly in the IH, suggesting functional plasticity of the muscle across the lifespan [27].

Our results on the LPM–MA relationship align with and expand upon the recent radiological studies by Albu et al. [6] and Piagkou et al. [1]. Albu et al. [6] reported that the MA most commonly coursed lateral/superficial to the LPM (63.2%). In comparison, a medial/deep trajectory (35.3%) and intramuscular penetration (1.5%) were less frequent, with significant correlations between specific LPM morphologies [6]. In contrast to the current findings, they observed a significant correlation between the MA intramuscular course and the LPM single head morphology [6]. In the current study, we did not observe any case of intramuscular course in one-headed LPM. To the authors’ knowledge, our finding is reasonable because an intramuscular course is possible when the MA courses between the muscular heads. Similarly, Piagkou et al. [1] found a predominance of the lateral course (64.2%), followed by medial (29.6%) and intramuscular (6.2%) variants, with pooled meta-analysis confirming the lateral trajectory as the most common (79.6%) but highlighting higher detection of medial and intramuscular courses in imaging-based cohorts [1]. Our CTA-based findings corroborate these observations, demonstrating that while the MA usually runs lateral to the LPM, medial and intramuscular variants are not negligible and may co-occur with complex multi-headed morphologies.

Innervation adds another layer of complexity. V3 branches mainly supply the LPM, but 3D reconstructions demonstrate distinct neural inputs to the SH and IH from the buccal, deep temporal, and masseteric nerves [34]. Akita et al. [2] showed that even within a single “head,” separate bundles may correspond to discrete nerve twigs, supporting functional compartmentalization.

### 4.2. Clinical and Surgical Considerations

The LPM plays a fundamental role in mandibular kinematics and TMJ biomechanics, and its variability has major clinical implications. The IH assists mandibular opening and protrusion, whereas the SH stabilizes the disk–condyle complex during closing and clenching [35]. Variability in head number and insertion may influence disk–condyle coordination. Aberrant slips preferentially inserting into the disk could predispose to internal derangements, while asymmetrical distributions may contribute to laterality and chronic myofascial pain [32]. Electromyographic and MRI evidence confirm that SH morphology is associated with anterior disk displacement and TMJ dysfunction [9].

The intimate relationship between the LPM and V3 branches means that accessory bundles or hypertrophy may compress adjacent nerves, producing neuralgia or paresthesia [36]. Recent ultrasound-guided anatomical studies improve block accuracy by visualizing individual LPM slips, highlighting the role of imaging-guided anesthesia [37].

The ITF remains a challenging surgical corridor. Variability in LPM head number and the course of the MA complicates infratemporal and transantral procedures, raising hemorrhagic risks [1,6]. Finite element analysis shows that asymmetry or hypertrophy of the LPM can alter TMJ loading patterns, further influencing surgical outcomes [10].

Endoscopic skull base and TMJ surgeries require precise navigation, where aberrant LPM morphologies may obscure safe landmarks. Advanced imaging classifications and preoperative mapping are essential [6].

For regional anesthesia, LPM slips and MA variants may mislead traditional landmarks, explaining failed mandibular blocks. CT- or ultrasound-guided techniques are now advocated, and navigation-assisted botulinum toxin-A injections into the LPM have shown promising outcomes in refractory myogenic TMJ disorders [38].

Finally, LPM variability impacts prosthodontic and orthodontic outcomes, as asymmetry may modify mandibular kinematics and occlusal rehabilitation [4]. Its intimate vascular associations must also be respected in reconstructive microsurgery, particularly when the MA is harvested as a donor vessel. Emerging functional research also suggests roles for the LPM in speech coordination and orofacial rehabilitation beyond mastication [11].

### 4.3. Limitations

This study has several limitations. First, its retrospective design and reliance on CTA, while advantageous for vascular and osseous detail, provide limited soft-tissue resolution compared with MRI, which may have led to underestimation of subtle muscular subdivisions. Second, although the sample size was relatively large (250 patients, 500 sides), it was restricted to a single tertiary center in Greece, limiting generalizability; this is particularly relevant as our meta-analysis revealed population differences, with a significantly higher prevalence of one-headed morphologies in European cohorts. Third, the systematic review and meta-analysis included studies with considerable methodological heterogeneity, such as differences in dissection protocols, imaging modalities, and definitions of “heads” versus “slips,” which contributed to substantial statistical heterogeneity (I^2^ > 70%). However, this is common in anatomical meta-analysis. In addition, many studies had small sample sizes, which reduced precision. However, DOI plots showed no evidence of small-study effects. Finally, our dataset did not directly assess functional or pathological correlations. At the same time, MRI and histological evidence suggest associations between head morphology, disk displacement, and degeneration; CTA alone cannot substantiate these links. Future multimodal investigations integrating imaging, histology, biomechanics, and clinical outcomes are needed to overcome these limitations and better elucidate the functional impact of LPM variability.

## 5. Conclusions

The current CTA analysis and systematic review with meta-analysis confirm that while the two-headed form predominates (~74%), a substantial proportion of individuals present with one-, three-, or rarely four-headed variants, with the one-headed morphology occurring more frequently in European populations. CTA proved to be a reliable method for assessing these variations, and the pooled prevalence estimates confirmed the predominance of the two-headed form across populations. Recognition of these morphological patterns is essential for accurate anatomical understanding and has direct clinical relevance in relation to temporomandibular joint biomechanics and surgical planning.

## Figures and Tables

**Table 1:** Descriptive analysis of the lateral pterygoid muscle (LPM) heads regarding sides and sex distribution.

**LPM Morphology**	**Total** **(*n* = 500)**	**Left** **(*n* = 250)**	**Right** **(*n* = 250)**	***p*-Value**	**Males** **(*n* = 272)**	**Females** **(*n* = 228)**	***p*-Value**
Two-Headed	372 (74.4%)	188 (75.2%)	184 (73.6%)	0.555	192 (70.6%)	180 (78.9%)	0.095
Three-Headed	70 (14%)	36 (14.4%)	34 (13.6%)		48 (17.6%)	22 (9.7%)	
One-Headed	54 (10.8%)	26 (10.4%)	24 (11.2%)		30 (11%)	24 (10.5%)	
Four-Headed	4 (0.8)	0 (0%)	4 (1.6%)		2 (0.7%)	2 (1.8%)	

**Table 2:** Characteristics of the studies included in the systematic review and meta-analysis, including population, study type, number of sides analyzed, and risk of bias assessment according to the Anatomical Quality Assurance (AQUA) tool (Henry et al. 2017) [14].

**Study**	**Year**	**Population**	**Type of Study**	**Sides**	**Risk of Bias**
Cho and Lee [16]	1972	Asia	Cadaveric	70	Low
Sugisaki et al. [17]	1986	Asia	Cadaveric	26	High
Naohara et al. [18]	1989	Asia	Cadaveric	20	High
Wilkinson and Chan [19]	1989	America	Cadaveric	5	High
Abe et al. [20]	1993	Asia	Cadaveric	79	Low
Foucart et al. [21]	1998	Europe	Cadaveric	22	High
Fujita et al. [22]	2001	Asia	Cadaveric	20	Low
Akita et al. [23]	2003	Asia	Cadaveric	45	Low
Akar et al. [24]	2009	Asia	Cadaveric	50	Low
Filho et al. [25]	2009	America	Imaging (MRI)	178	Low
Kilic et al. [26]	2010	Asia	Cadaveric	49	Low
Abe et al. [27]	2011	Asia	Cadaveric	30	High
Antonopoulou et al. [8]	2013	Europe	Cadaveric	36	Low
Villar et al. [28]	2021	America	Cadaveric	30	Low
Albu et al. [6]	2025	Europe	Imaging (CT, CTA, CBCT)	542	Low
Current Study	2025	Europe	Imaging (CTA)	500	-

**Table 3:** Subgroup analysis of the pooled prevalence of lateral pterygoid muscle (LPM) morphologies (two-headed, three-headed, and one-headed) based on geographical distribution, study type, and sample size. Data are displayed as pooled frequencies (%) from random-effects meta-analysis. *p* < 0.05 is considered statistically significant. A significant difference was observed in one-headed prevalence by geographical distribution (*p* < 0.001), with a higher frequency in European studies.

**Parameters**	**Two-Headed**	**Three-Headed**	**One-Headed**
Asia (n = 9)	73.51	16.54	5.06
Europe (n = 4)	69.96	17.35	8.83
America (n = 3)	81.64	18.36	0.00
*p*-value	0.1227	0.8197	<0.0001
Cadaveric (n = 13)	74.25	16.00	4.41
Imaging (n = 3)	72.41	20.19	4.97
*p*-value	0.8783	0.6297	0.8140
Sample over 50 muscles (n = 6)	74.29	16.94	5.32
Sample under 50 muscles (n = 10)	73.54	17.07	4.01
*p*-value	0.7640	0.8173	0.9412

## Data Availability

All the data are available upon reasonable request to the authors.

## References

[1] Piagkou M., Triantafyllou G., Papadopoulos-Manolarakis P., Demetriou F., Tsakotos G., Olewnik Ł., Duparc F. (2025). Mapping the Maxillary Artery and Lateral Pterygoid Muscle Relationship: Insights from Radiological and Meta-Analytic Evidence. Medicina.

[2] Akita K., Sakaguchi-Kuma T., Fukino K., Ono T. (2019). Masticatory Muscles and Branches of Mandibular Nerve: Positional Relationships Between Various Muscle Bundles and Their Innervating Branches. Anat. Rec..

[3] Triantafyllou G., Papadopoulos-Manolarakis P., Luzzi S., Olewnik Ł., Tsakotos G., Zielinska N., Galzio R., Tudose R.C., Rusu M.C., Piagkou M. (2025). Foramen ovale morphology and relationship with the lateral pterygoid process plate: Proposal for a new classification system. Anat. Sci. Int..

[4] Stöckle M., Fanghänel J., Knüttel H., Alamanos C., Behr M. (2019). The morphological variations of the lateral pterygoid muscle: A systematic review. Ann. Anat.-Anat. Anz..

[5] Standring S. (2016). Gray’s Anatomy: The Anatomical Basis of Clinical Practice.

[6] Albu A.C., Tudose R.C., Vrapciu A.D., Rusu M.C. (2025). Beyond two heads: An imaging-based analysis of the lateral pterygoid muscle’s heads. Ann. Anat.-Anat. Anz..

[7] Usui A., Akita K., Yamaguchi K. (2008). An anatomic study of the divisions of the lateral pterygoid muscle based on the findings of the origins and insertions. Surg. Radiol. Anat..

[8] Antonopoulou M., Iatrou I., Paraschos A., Anagnostopoulou S. (2013). Variations of the attachment of the superior head of human lateral pterygoid muscle. J. Cranio-Maxillofac. Surg..

[9] Melke G.S.d.F., Costa A.L.F., Lopes S.L.P.d.C., Fuziy A., Ferreira-Santos R.I. (2016). Three-dimensional lateral pterygoid muscle volume: MRI analyses with insertion patterns correlation. Ann. Anat.-Anat. Anz..

[10] Yang C., Teng H., Shao B., Liu Z. (2024). Biomechanical study of temporomandibular joints of patients with temporomandibular disorders under incisal clenching: A finite element analysis. J. Biomech..

[11] Murray G.M., Orfanos T., Chan J.Y., Wanigaratne K., Klineberg I.J. (1999). Electromyographic activity of the human lateral pterygoid muscle during contralateral and protrusive jaw movements. Arch. Oral Biol..

[12] Henry B.M., Tomaszewski K.A., Walocha J.A. (2016). Methods of Evidence-Based Anatomy: A guide to conducting systematic reviews and meta-analysis of anatomical studies. Ann. Anat..

[13] Page M.J., McKenzie J.E., Bossuyt P.M., Boutron I., Hoffmann T.C., Mulrow C.D., Shamseer L., Tetzlaff J.M., Akl E.A., Brennan S.E. (2021). The PRISMA 2020 statement: An updated guideline for reporting systematic reviews. BMJ.

[14] Henry B.M., Tomaszewski K.A., Ramakrishnan P.K., Roy J., Vikse J., Loukas M., Tubbs R.S., Walocha J.A. (2017). Development of the anatomical quality assessment (AQUA) tool for the quality assessment of anatomical studies included in meta-analyses and systematic reviews. Clin. Anat..

[15] Furuya-Kanamori L., Barendregt J.J., Doi S.A.R. (2018). A new improved graphical and quantitative method for detecting bias in meta-analysis. Int. J. Evid. Based Healthc..

[16] Cho H.J., Lee Y.S. (1972). A variety of number of heads of external pterygoid muscle. Taehan Chikkwa Uisa Hyophoe Chi.

[17] Sugisaki M., Komori E., Nakazawa M., Tanabe H., Kato S. (1986). Anatomical studies of the lateral pterygoid muscle by the superior approach and a review of the literature. Jpn. J. Oral Maxillofac. Surg..

[18] Naohara H. (1989). The macroscopic and microscopic study of the human lateral pterygoid muscle. Tsurumi Shigaku.

[19] Wilkinson T., Chan E.K.K. (1989). The anatomic relationship of the insertion of the superior lateral pterygoid muscle to the articular disc in the temporomandibular joint of human cadavers. Aust. Dent. J..

[20] Abe S., Takasaki I., Ichikawa K., Ide Y. (1993). Investigations of the run and the attachment of the lateral pterygoid muscle in Japanese. Bull. Tokyo Dent. Coll..

[21] Foucart J.M., Girin J.P., Carpentier P. (1998). Innervation of the human lateral pterygoid muscle. Surg. Radiol. Anat..

[22] Fujita S., Iizuka T., Dauber W. (2001). Variation of heads of lateral pterygoid muscle and morphology of articular disc of human temporomandibular joint—Anatomical and histological analysis. J. Oral Rehabil..

[23] Akita K., Shimokawa T., Sato T. (2003). An anatomic study of the positional relationships between the lateral pterygoid muscle and its surrounding nerves. Eur. J. Anat..

[24] Coskun Akar G., Govsa F., Ozgur Z. (2009). Examination of the Heads of the Lateral Pterygoid Muscle on the Temporomandibular Joint. J. Craniofacial Surg..

[25] Filho H.P., Guimarães A.S., Galdames I.C.S. (2009). Prevalence of the Third Head of the Lateral Pterygoid Muscle: A Magnetic Resonance Image Study. Int. J. Morphol..

[26] Kiliç C., Dergïn G., Yazar F., Kurt B., Kutoğlu T., Ozan H., Balcioğlu H.A. (2010). Insertions of the lateral pterygoid muscle to the disc-capsule complex of the temporomandibular joint and condyle. Turk. J. Med. Sci..

[27] Abe S., Naito H., Nakao T., Yamamoto M., Won S.-Y., Kim H.-J., Ide Y. (2011). Analysis of the Intramuscular Innervation of the Lateral Pterygoid Muscle. J. Hard Tissue Biol..

[28] Villar C.T., Deana N.F., Sousa-Rodrigues C.F., Alves N. (2021). Evaluation of the Presence of the Third Head on the Lateral Pterygoid Muscle in Adult Individuals. Int. J. Morphol..

[29] Katori Y., Yamamoto M., Asakawa S., Maki H., Rodríguez-Vázquez J.F., Murakami G., Abe S. (2012). Fetal developmental change in topographical relationship between the human lateral pterygoid muscle and buccal nerve. J. Anat..

[30] Rusu M.C., Toader C., Tudose R.C., Grigoriţă L.O. (2024). Debate on the Morphological Variability of the Lateral Pterygoid Muscle—Discrepancies, Speculations and New Original Anatomical Samples. Medicina.

[31] Mandal G., Montalbano M., Natsis K., Piagkou M., Tubbs R.S., Loukas M. (2023). Musculus pterygoideus proprius: A meta-analysis. Clin. Anat..

[32] Rusu M.C., Stoenescu M.D., Săndulescu M. (2024). The Maxillomandibularis Muscle. J. Craniofacial Surg..

[33] Monemi M., Thornell L.-E., Eriksson P.-O. (1999). Diverse changes in fibre type composition of the human lateral pterygoid and digastric muscles during aging. J. Neurol. Sci..

[34] Davies J., Charles M., Cantelmi D., Liebgott B., Ravichandiran M., Ravichandiran K., Agur A. (2012). Lateral pterygoid muscle: A three-dimensional analysis of neuromuscular partitioning. Clin. Anat..

[35] McNamara J.A. (1973). The independent functions of the two heads of the lateral pterygoid muscle. Am. J. Anat..

[36] Piagkou M.N., Demesticha T., Piagkos G., Androutsos G., Skandalakis P. (2011). Mandibular nerve entrapment in the infratemporal fossa. Surg. Radiol. Anat..

[37] Rodríguez-Gimillo P., Valverde-Navarro A., Margaix-Muñoz M., Poveda-Roda R., Delgado-Navarro C., Puig-Bernabeu J. (2024). Lateral pterygoid muscle ultrasound-guided injection: A technical note. J. Stomatol. Oral Maxillofac. Surg..

[38] Martenot A., Devoti J.-F., Pons M., Meyer C., Brumpt E., Louvrier A., Bertin E. (2024). Persistent myogenic temporomandibular disorders: Are navigation-guided botulinum toxin-A injections into the lateral pterygoid muscles effective?. J. Stomatol. Oral Maxillofac. Surg..

